# The Cost-Effectiveness Analysis of Self-Efficacy-Focused Structured Education Program for Patients With Type 2 Diabetes Mellitus in Mainland China Setting

**DOI:** 10.3389/fpubh.2021.767123

**Published:** 2021-12-09

**Authors:** Xinjun Jiang, Hua Jiang, Libo Tao, Mingzi Li

**Affiliations:** ^1^Department of Adults Nursing, School of International Nursing, Hainan Medical University, Haikou, China; ^2^Department of Medical and Surgical Nursing, School of Nursing, Peking University, Beijing, China; ^3^Key Laboratory of Emergency and Trauma Ministry of Education, Hainan Medical University, Haikou, China; ^4^Key Laboratory of Trauma of Hainan Medical University, Hainan Medical University, Haikou, China; ^5^Key Laboratory of Tropical Cardiovascular Diseases Research of Hainan Province, The First Affiliated Hospital of Hainan Medical University, Haikou, China; ^6^Center for Health Policy and Technology Evaluation, Peking University Health Science Center, Beijing, China

**Keywords:** diabetes mellitus, type 2, self-efficacy, structured education, cost-effectiveness analysis, economic evaluation

## Abstract

**Objective:** To assess the long-term (50 years) cost-effectiveness of the self-efficacy-focused structured education program (SSEP) as opposed to routine education among patients with type 2 diabetes mellitus (T2DM) in mainland China from a healthcare service perspective.

**Methods:** A cost-effectiveness analysis method was used. The IQVIA CORE Diabetes Model (version 9.0) was adopted to estimate the outcomes. The baseline cohort characteristics, variations of physiological parameters, costs of intervention and other treatments, and management-related diabetes were derived from a randomized controlled trial. Moreover, the complications costs and utilities were extracted from published sources. Furthermore, the univariate sensitivity analysis and the probabilistic sensitivity analysis were conducted.

**Results:** As compared with the control group, the life expectancy and quality-adjusted life-year in the intervention group were increased. Besides, the intervention group achieved lower cumulative incidences of complications and saved more direct medical costs compared with the control group. The sensitivity analysis revealed that the SSEP had 100% probability to be cost-effective.

**Conclusion:** The SSEP is recognized as a highly cost-effective option for managing patients with T2DM, which are projected to both improve clinical outcomes and reduce costs.

## Introduction

By 2019, diabetes mellitus had affected ~9.3% of adults worldwide, nearly 80% of whom lived in low- and middle-income nations (1). For China, one of the biggest middle-income nations, the issue imposed by diabetes is noticeably severe. As indicated from the latest national investigation in 2017, the prevalence of diabetes in the whole nation, the urban and rural areas, was 12.8, 13.7, and 12.0%, respectively, in mainland China according to the American Diabetes Association (ADA) diagnosis criteria; in addition, the increase in the current prevalence of diabetes in the rural area was 2.5 times that of the urban area (2). Among all the diabetes patients, type 2 diabetes mellitus (T2DM) is nearly 90 to 95%. Diabetes and its complications (e.g., cardiovascular diseases, renal diseases, and retinopathy) will cause high morbidity and premature death and impose a heavy economic burden on the healthcare systems and national economies. By 2019, the total diabetes-related cost was estimated as 760 billion US dollars worldwide and 109 billion US dollars in China, taking up about 12.65% of the overall health expenditure in mainland China (1, 3). However, the costs associated with diabetes and its complications and even the morbidity and premature death can be downregulated through effective management. The effective management of T2DM is dependent on medication and the non-drug intervention (e.g., diabetes education) that aims to facilitate the daily self-management of patients (e.g., appropriate diet, regular physical activity, and psychological well-being). However, the economic evaluation of the non-drug intervention (e.g., diabetes education) should be still conducted due to the limited health resources.

Diabetes structured education, as one of the satisfactory diabetes educations, has been set out as a priority by the international and some national guidelines for the purpose of helping patients with diabetes mellitus that develop self-management behaviors (4–6). Diabetes structured education has been defined as the education based on psychological and treatment needs, characteristics of the patients have been considered, and the education has been delivered with a plan and stratification for patients with diabetes (7). The short-term effectiveness exhibited by the diabetes structured education program was confirmed, i.e., it is capable of promoting self-management behavior changes and improving metabolic control (e.g., glycemic control) in patients with T2DM (8, 9). Meanwhile, the economic evaluation of the structured education program conducted in patients with T2DM by some scholars outside mainland China has been shown the cost-effectiveness of the program, which achieved quality-adjusted life-years and cost increased at the accessible threshold range (10–13). However, one of them is aimed at evaluating the short-term cost-effectiveness of the structured education program (13) and most studies are less comprehensive in considering the cost or predicting diabetes-related complications (10–12). In addition, diabetes structured education is in the initial stage in many developing countries including China (14, 15) and the rigorous long-term cost-effectiveness analysis is scarce. Furthermore, the WHO (16) suggested that whether a program/intervention is cost-effective that is determined by the actual circumstances of each nation/region due to the uneven level of economic development. Subsequently, a structured education program is cost-effective in developing countries, especially in mainland China, should be assessed in depth.

The self-efficacy-focused structured education program (SSEP) for patients with T2DM with noninsulin therapy developed by Liu et al. (17) is a well-designed diabetes structured education program in mainland China that accords with the principles of structured education program recommended by the National Institute for Health and Care Excellence. In addition, the program also incorporates self-efficacy theory, cultural characteristics of Chinese, and others (18). Then, a multicenter randomized controlled trial (RCT) was conducted to assess the clinical effectiveness of the SSEP. The findings indicated that the effect on glycemic control, diabetes self-management behaviors, and psychosocial aspects were positive at 6-month follow-up (18) and the mentioned encouraging effects could sustain at 12-month follow-up (19, 20). Nevertheless, the SSEP did not receive the economic assessment that limited its wide-scale implementation in mainland China. For this reason, this study aimed to assess the long-term cost-effectiveness of the SSEP.

## Materials and Methods

### Analytical Method

A cost-effectiveness analysis model was adopted. The cost-effectiveness analysis was mainly conducted based on a multicenter RCT conducted in mainland China. The RCT was approved by the Review Board of Peking University (IRB00001052-17031) and registered in the Chinese Clinical Trial Registry (ChiCTR-IOR-17011007). The IQVIA CORE Diabetes Model (version 9.0) was adopted to simulate the long-term results.

### Model Description

Since the lifetime follow-up of the participants in this study has been rarely feasible, the long-term cost-effectiveness analysis of the SSEP was performed in comparison with routine education from a healthcare service perspective using a peer-reviewed, transparent, and validated computer simulation model of the IQVIA CORE Diabetes Model (21, 22) (CDM, www.core-diabetes.com). The credibility of the model examined in 2004 and more recently in 2014 was satisfactory (21, 22). Currently, the model has also been applied for Chinese patients with diabetes (23). CDM was developed by Palmer et al. (24) and the whole structure, characteristics, and function of the model were discussed and reported in the published literature previously (21, 25). CDM involves 17 submodel of diabetes complications including angina, myocardial infraction, congestive heart failure, stroke, peripheral vascular disease, foot disease, retinopathy, nephropathy, neuropathy, macular edema, cataract, hypoglycemia, ketoacidosis, lactic acidosis, edema, nonspecific mortality, and depression. Adopting Markov techniques, each of the submodel relied on Monte Carlo simulations to project the primary outcomes of quality-adjusted life expectancy [as displayed with quality-adjusted life-years (QALYs)], directed medical costs and the incremental cost-effectiveness ratios (ICERs) (a core indicator in cost-effectiveness analysis as calculated using incremental costs and gains in QALYs in the CDM), the secondary outcome of diabetes complications, and life expectancy. The Markov models incorporate a set of health states and the transition probabilities between different states of the model. For example, the Markov model tree intended for submodel in the CDM involves three different states (healthy, diseased, and dead). A subject in a healthy state might maintain that state, shift to developing disease, or die. Additionally, the diseased state can progress either into healthy state or dead state, with dead as the terminal state. As for Monte Carlo simulation, it can overcome the memory-less characteristics of the Markov model by allowing the interaction between each complication of submodel. With respect to such model input data as baseline cohort characteristics, the variations of physiological parameters, the costs of intervention and other treatments, and management-related diabetes, they were obtained from a multicenter RCT. The complications costs and utilities were extracted from published sources due to no primary data available in the practical multicenter RCT. Moreover, some physiological parameters and the progression of all the physiological parameters were determined through the UK Prospective Diabetes Study (UKPDS) and the Framingham study. A nonparametric bootstrapping approach was adopted in the model to perform simulation 1,000 times with a cohort of 1,000 nonidentical patients that sampled from the baseline cohort. As to distributions of parameters, they mainly conformed to a normal distribution, Weibull distribution, gamma distributions, etc. The details could be found in the published literature (21, 22). In addition, when conducting the simulation, clinical effect and cost were downregulated at a rate of 3.5% annually by following related health economic guidance in China (26) and the outcomes were projected for a lifetime (50 years) given the mean age of the population.

### Major Assumptions

a. The parameters used in the IQVIA CORE Diabetes Model were obtained from the western population. It was assumed that these parameters were conformed to the characteristics of the Chinese population in this study.
b. The utilities were extracted from the published literature. It was assumed that they were suitable for the targeted population participating in this study.
c. The cost of complications was determined according to the previous studies conducted in China. It was assumed that they were applicable to the population involved in this study.

### Participants and Intervention

The population and interventions were set according to a RCT reported in the previous published publications (18, 20), the population was patients with T2DM with noninsulin therapy, and the participants in this study received the SSEP or routine education. The trial is given in Table 1.

**Table 1:** Overview of the trial.

	**Jiang et al. (19); Jiang et al. (20)**
Setting	Four hospitals in mainland China
Participants	T2DM patients with noninsulin therapy
Interventions	SSEP: The structured education program that was self-efficacy-focused and culture sensitive was comprised of four weekly modules (60–90 min each). And the program was delivered in a group format of 4–8 patients to allow the interaction between the educator and patients. Routine education: Individual based counselling by physicians and didactic class education about diabetes by physicians or nurses.
Length of study	12-month follow-up study period
Number of participants	265
Outcomes	The metabolic outcomes of HbA1c, weight, body mass index, waist circumference, blood pressure, and blood lipid profile, and the psychosocial outcomes of diabetes-related knowledge, self-efficacy, self-management behaviors and diabetes distress.

### Model Inputs

#### Clinical Data

Clinical data to inform the cost-effectiveness analysis were extracted from the practical multicenter RCT reported by Jiang et al. (18–20). Baseline cohort characteristics (e.g., demographics characteristics, baseline risk factors, and complications events history) were adopted from this study. The mean (SD) age and duration of T2DM were 56.91 (10.05) and 6.03 (5.17) years, respectively. The proportion of males was 44.9%. The mean hemoglobin A1c (HbA1c) was 8.71 (1.35)% and other baseline cohort characteristics are shown in Appendix S1. The clinical effect of the physiological parameters with an intervention group (received the SSEP) and control group (received routine education commonly presented by the clinics) were determined by the mean changes from baseline and 12-month follow-up multicenter RCT (Table 2) according to the confounding factors controlled (e.g., demographic information, diabetes-related information, comorbidities, and diabetes medication use). In addition, diabetes medication use adjustments during the 12-month follow-up between the two groups were compared and the results showed no significance (19, 20). The hypoglycemic events were also collected in this study (Table 2) (19, 20). Furthermore, some other physiological parameters and the transition probabilities of the physiological parameters were modeled with the default setting of the CDM.

**Table 2:** Changes in clinical effects (12 months).

**Parameter**	**Mean (standard error)**	**Mean (standard error)**
	**Intervention group**	**Control group**
HbA1c, %	−1.595 (0.140)	−0.693 (0.140)
Systolic pressure, mmHg	−5.535 (1.149)	−1.886 (1.153)
Diastolic pressure, mmHg	−4.368 (0.772)	−3.015 (0.775)
TC, mmol/L	−0.546 (0.119)	−0.165 (0.119)
LDL, mmol/L	−0.219 (0.084)	−0.036 (0.084)
HDL, mmol/L	−0.022 (0.054)	−0.042 (0.054)
TG, mmol/L	−0.423 (0.117)	−0.223 (0.118)
BMI, kg/m^2^	−0.446 (0.171)	+0.179 (0.172)
Waist hip ratio	−0.009 (0.005)	+0.002 (0.005)
Non-severe hypoglycemic event rate (events per 100 per year)	0	+15.9

*TC, total cholesterol; TG, triglycerides; LDL-C, low-density lipoprotein cholesterol; HDL-C, high-density lipoprotein cholesterol; BMI, body mass index*.

#### Costs and Utilities

The analysis was conducted on the perspective of the healthcare service system in mainland China setting, with all the costs adjusted in consumer price index level in China in 2017. The patients that participated in the 12-month follow-up RCT did not pay for any costs, e.g., fee for the SSEP, but indeed, the SSEP consumed resources, so, the cost of the SSEP was calculated in this study. The cost of the SSEP included salary costs (the cost of trainers and trainees, which included nurses and physicians), materials costs, delivering costs, operational costs, transportation costs, and other related costs. The cost of the SSEP for each patient is 237.52 renminbi (RMB) after calculation through the actual cost records by the researcher. The costs of the program after the first year were the expenses for follow-up of the patients, i.e., 35.67 RMB per patient in the subsequent year.

The cost of the treatment for patients was collected by the research nurses in the respective research center using unified self-designed questionnaires at 3-, 6-, and 12-month follow-ups. The self-designed questionnaires included the biochemical examination record questionnaire, antihyperglycemic agent record questionnaire, and the patient medical cost questionnaire. The biochemical examination record questionnaire was used to collect the test status of HbA1c, fasting blood glucose, and blood lipid profile. The antihyperglycemic agent record questionnaire was adopted to obtain the medicine use, status of metformin, insulin-secreting agents, glycosidase inhibitors, dipeptidyl peptidase-4 (DPP-4) inhibitors, and thiazolidinediones, etc. The medical cost questionnaire of the patient was chosen to get the cost of hospitalization, self-monitoring, other medication management, and screening. To ensure the accuracy of the data, the costs of the biochemical test, hospitalization, and screening were collected through the payment bill by the patients combined with the electronic medical system. The cost of the antihyperglycemic agent, other medicine usages, and self-monitoring were calculated based on the dosage/monitoring frequency in which the questionnaire recorded multiplied by the latest retail prices in China in 2018, respectively. The costs of treatment for the intervention group saved 381.83 RMB per patient as compared with that in the control group after calculation. As the disease progresses, most patients develop diabetes complications that affect their holistic health-related quality of life. The cost of treating diabetes complications in the year of the event and in the subsequent year was determined based on the literature (Appendix S2). All the costs inputted in the model were regulated by adopting the consumer price index for health in China. The utilities applied in the analysis model were primarily obtained from a review by Beaudet et al. (27) and some other published sources (28–30). The values of utilities in each diabetes complication state are shown in Appendix S3.

#### Sensitivity Analyses

The sensitivity analyses were conducted by performing the key parameters to identify the robustness of the base case results. The methods of the univariate sensitivity analysis and the probabilistic sensitivity analysis (PSA) were used. To determine the effect of cost, clinical effect, and utilities on the results of the analyses, the costs of the SSEP, the costs of diabetes complications, the difference of the clinical effect between the two groups, and utilities fluctuated ±20%, respectively. Ten years, 20 years, and 30 years were assumed to determine the effect of time horizon on the results. The discount rate (i.e., 0, 6, and 8%) was set to assess the uncertainty of the simulated results. Moreover, the PSA was performed with nonparametric bootstrapping methods. The data of demographic characteristics, utilities, and the clinical effect were selected from distributions and the data of cost fluctuated ±10% at the means value. Furthermore, the cohort of 1,000 nonidentical patients was simulated 1,000 times. The results were illustrated as the scatterplot graph, tornado diagrams, and acceptability curve.

## Results

### Base Case Analysis

The 50-year simulation of clinical outcomes indicated that the cumulative incidence of diabetes-related complications of cardiovascular diseases, renal diseases, eye diseases, diabetic foot complications, neuropathy, hypoglycemia, and mortality due to diabetes mellitus in the intervention group was 7.75, 2.16, 2.01, 5.27, 1, 20.22, and 0.98%, respectively lower than those in the control group (Appendix S4). The life expectancy and QALYs of per patient in the intervention group were gained more than those in the control group (14.832 ± 0.164 vs. 14.632 ± 0.163 years; 10.396 ± 0.115 vs. 10.052 ± 0.112 QALYs) (Table 3).

**Table 3:** Long-term cost-effectiveness outcomes (50 years).

	**Intervention group Mean (SD)**	**Control group** **Mean (SD)**	**Difference**
Discounted life expectancy, years	14.832 (0.164)	14.632 (0.163)	0.200
Discounted quality-adjusted life-years, QALYs	10.396 (0.115)	10.052 (0.112)	0.344
Discounted direct medical cost, RMB	299764 (5298)	334229 (5858)	−34465
ICER based on life expectancy and direct medical cost	SSEP dominant		
ICER based on quality-adjusted life-years and direct medical cost	SSEP dominant		

*QALYs, quality-adjusted life-years; RMB, renminbi; ICER, incremental cost-effectiveness ratio*.

The result of direct medical costs indicated that there was 34,465 RMB per patient saved in the intervention group compared to the control group. The cost-saving was predominantly by lower cost of diabetes-related complications and cost of the intervention and other treatment (Appendix S5).

The intervention group having received the SSEP was associated with long-term clinical outcomes improvements of diabetes-related complications, life expectancy, and QALYs. Meantime, the mean cost per patient was lower in the intervention group. So, the SSEP was considered dominant over routine education and the ICER was not required calculating (Table 3).

### Sensitivity Analyses

**Figure 1:**
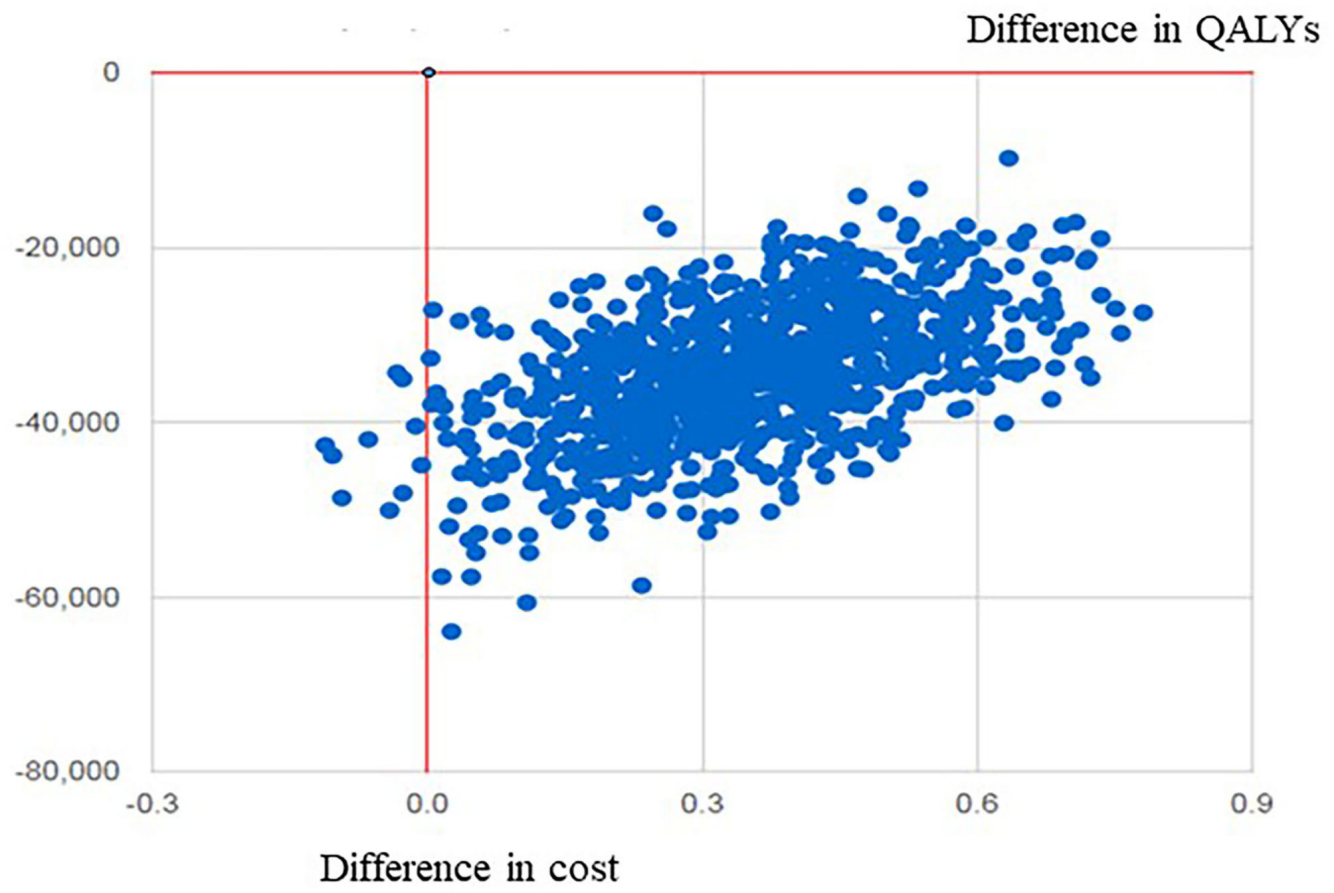
The scatter plot graph of cost-effectiveness of the SSEP compared with routine education. SSEP, self-efficacy-focused structured education program; QALYs, quality-adjusted life-years.

**Figure 2:**
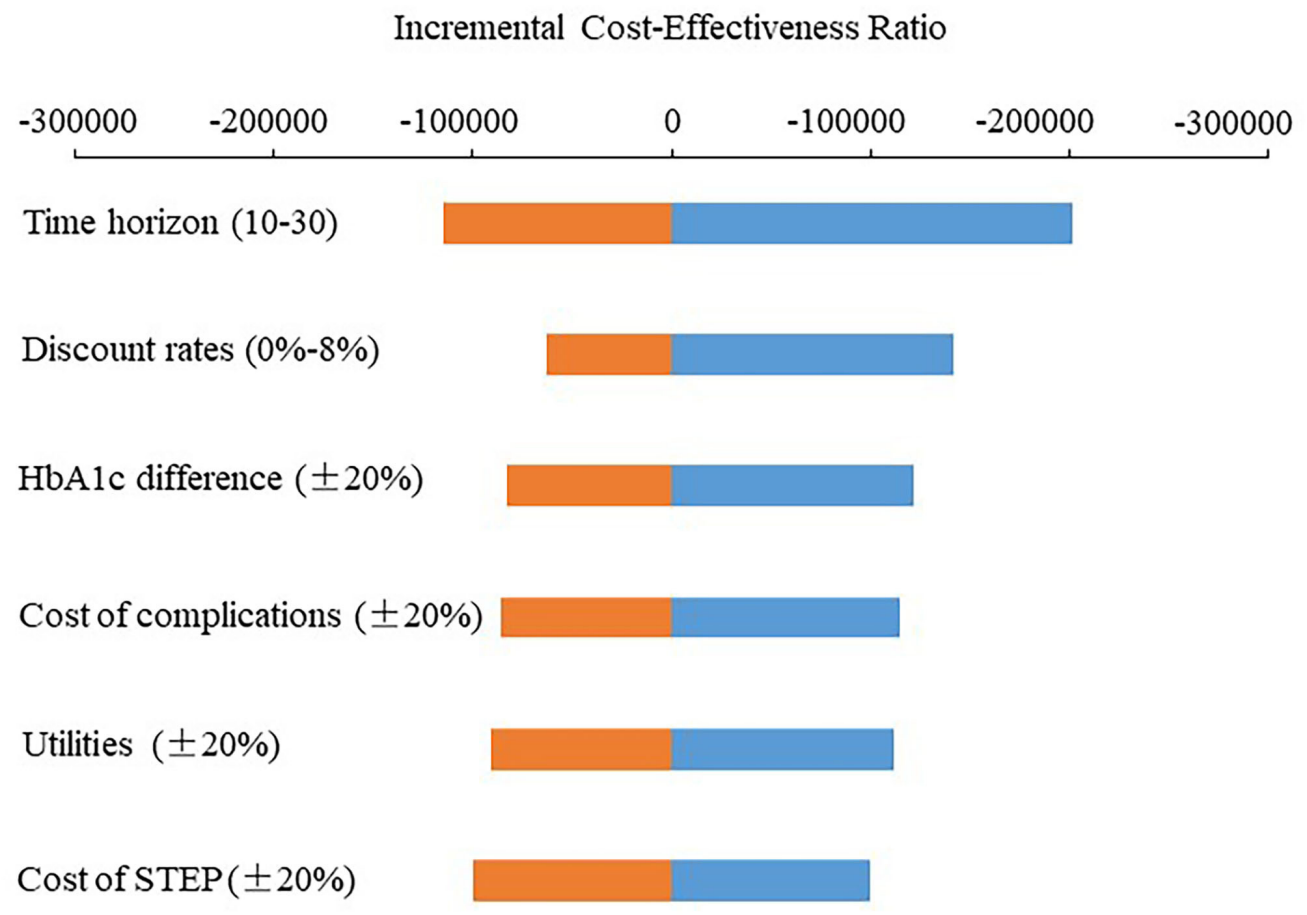
Tornado diagram of the SSEP compared with routine education.

**Figure 3:**
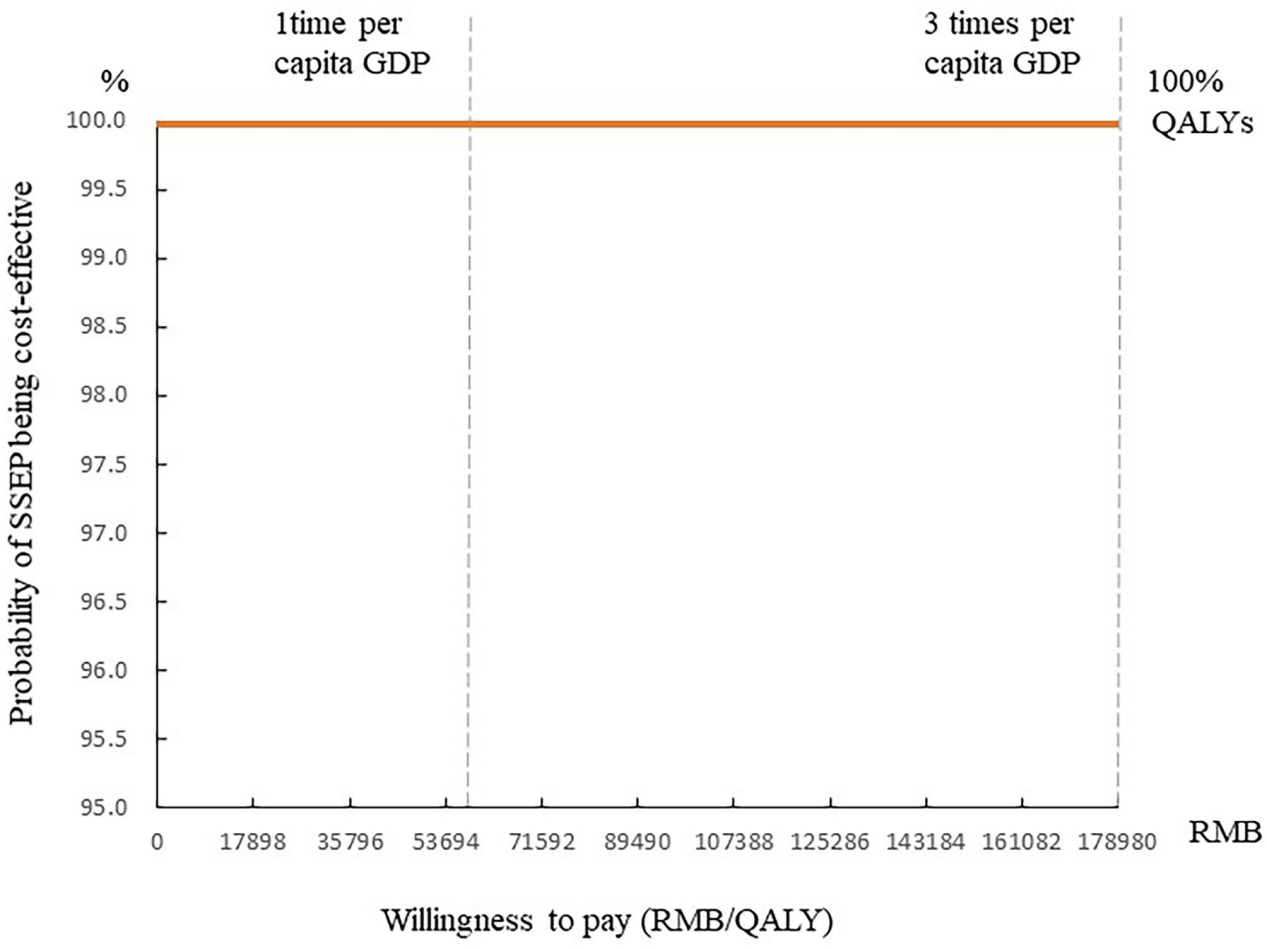
The cost-effectiveness acceptability curve of the SSEP. GDP, gross domestic product; QALYs, quality-adjusted life-years.

Table 4 lists the results of the univariate sensitivity analysis. Compared with the control group, the intervention group received the SSEP that can obtain more QALYs and result in cost-saving with the changes of the discount rate, time horizon, cost of the SSEP, cost of complications, HbA1c effect, and utility. It is, therefore, indicated that the SSEP dominates routine education (Table 4). Figure 1 presents the ICER scatter graph producing by the PSA. The horizontal axis and the vertical axis represent the difference in QALYs and cost between the two groups, respectively. The original point stands for routine education, while the scatter represents the SSEP. Figure 1 indicates that most of the scatters are concentrated in the fourth quadrant of the ICER scatter graph, which indicated that the SSEP is effective and cost-saving. Figure 2 also shows that the SSEP is cost-saving. In addition, the cost-effectiveness acceptability curve (Figure 3) reveals that the SSEP is cost-effectiveness at one time per capita gross domestic product (GDP) (59,660 RMB) or three times per capita GDP (178,980 RMB) in 2017 in mainland China. Moreover, there is ~100% probability that the SSEP would be cost-effective at the level of willingness to pay 59,660 RMB or 178,980 RMB per QALY threshold.

**Table 4:** Results of sensitivity analysis.

**Parameters of sensitivity analysis**	**Discounted quality-adjusted life expectancy (QALYs)**			**Discounted direct medical cost (RMB)**			
	**Intervention** **group Mean (SD)**	**Control group** **Mean (SD)**	**Difference**	**Intervention group** **Mean (SD)**	**Control group Mean (SD)**	**Difference**	**ICER (RMB/per QALY gained)**
Discount rates							
0%	15.887 ± 0.238	15.295 ± 0.232	0.592	541531.75 ± 11938.96	578733.63 ± 12828.39	−37201.91	−62841.06
6%	8.1450 ± 0.076	7.890 ± 0.074	0.256	210663.14 ± 3380.19	242507.47 ± 3808.98	−31844.33	−124391.91
8%	6.883 ± 0.057	6.674 ± 0.056	0.210	164322.80 ± 2517.34	194146.11 ± 2874.32	−29823.32	−142015.81
Time horizon							
10 year	5.864 ± 0.034	5.702 ± 0.034	0.161	101544.58 ± 1794.00	134082.41 ± 1940.38	−32537.81	−202098.20
20 year	8.936 ± 0.078	8.682 ± 0.076	0.254	217169.98 ± 3758.72	252228.48 ± 3648.82	−35058.50	−138025.59
30 year	10.079 ± 0.097	9.771 ± 0.100	0.308	278604.22 ± 5066.26	314081.88 ± 5328.83	−35477.66	−115187.21
Cost of SSEP							
+20%	10.396 ± 0.115	10.052 ± 0.112	0.344	299830.13 ± 5298.05	334228.75 ± 5857.87	−34398.60	−99995.93
−20%	10.396 ± 0.115	10.052 ± 0.112	0.344	299698.00 ± 5298.02	334228.75 ± 5857.87	−34530.72	−100380.00
Cost of Complications							
−20%	10.396 ± 0.115	10.052 ± 0.112	0.344	256858.05 ± 4376.09	286332.22 ± 4822.30	−29474.17	−85680.73
+20%	10.396 ± 0.115	10.052 ± 0.112	0.344	342670.13 ± 6224.58	382125.28 ± 6897.71	−39455.16	−114695.23
HbA1c difference							
−20%	10.339 ± 0.109	10.052 ± 0.112	0.287	310358.03 ± 5926.88	334228.75 ± 5857.87	−23870.70	−83173.17
+20%	10.422 ± 0.105	10.052 ± 0.112	0.371	288980.63 ± 5439.35	334228.75 ± 5857.87	−45248.10	−121962.53
Utilities							
+20%	12.540 ± 0.139	12.160 ± 0.136	0.380	299764.06 ± 5298.04	334228.75 ± 5857.87	−34464.66	−90696.47
−20%	8.251 ± 0.091	7.944 ± 0.089	0.307	299764.06 ± 5298.04	334228.75 ± 5857.87	−34464.66	−112262.74

## Discussion

As revealed from the principal findings of the simulation, the SSEP is effective and cost-saving over a 50-year time horizon in comparison with routine education and the simulation result is robust. To the best of our knowledge, it is the first study in mainland China to conduct the economic assessment of a structured education program for diabetes mellitus. This study would contribute evidence for the long-term clinical effect and economic efficiency of the SSEP and support the decision-making process for policymakers.

This study found that the SSEP was dominant over routine education improving long-term clinical outcomes and reducing direct medical cost in patients with T2DM with noninsulin therapy. Previously published studies found that though the structured education program was cost-effective for patients with T2DM, the improved clinical outcomes are usually accompanied with the increase of the cost (10–12, 31, 32). Gillett et al. found that the Diabetes Education and Self-management for Ongoing and Newly Diagnosed (DESMOND) program could help each patient gain extra 0.0392 QALYs, but the cost of the DESMOND program increased by £82 per person, so the ICER was £2092/QALY (12). The result of this study indicates that the SSEP is cost-saving. The satisfactory result might be mainly due to the significant improvement of metabolic outcomes of the patients after they received the SSEP, so that the cumulative incidences of complications, e.g., cardiovascular diseases, renal diseases, eye diseases, diabetic foot complications, and neuropathy in the intervention group, were lower than that in the control group. Thus, the increase in QALYs is the result of improved long-term health outcomes and cost-saving is mainly due to the reduction of diabetes-related complication treatment. Another important reason is that the implementation and follow-up of the SSEP were led by trained registered nurses that would contribute to a relatively low human cost. Furthermore, the expenses in the two groups (e.g., oral hypoglycemic drugs, hospitalization expenses) after the patients received the SSEP were considered, while other studies (10, 12) were not. The reduction of the costs mentioned in the intervention group could entirely offset the increased expenses of the SSEP.

The results of the univariate sensitivity analysis and the PSA demonstrated the robustness of the simulation. The SSEP is still dominant by altering the values of critical parameters (e.g., discount rate, time horizon, cost of the SSEP, cost of complications, HbA1c effect, and utility changes). Moreover, a probabilistic sensitivity analysis that is based on the ICER per QALY per patient gained manifested that the SSEP is nearly 100% probability of being cost-effective at a willingness to pay threshold of one-time per capita GDP per QALY or three-times per capita GDP per QALY in mainland China.

This study indicated that the SSEP is an effective and cost-saving intervention for patients with T2DM with noninsulin therapy, which supports its widespread implementation in mainland China. It would be conducive to saving a substantial cost for healthcare systems, if the SSEP can be incorporated into routine medical service of diabetes mellitus. But, the implementation of the SSEP has been exclusively determined by the selfless dedication of the medical staffs. The free education model may be difficult to replicate and will fritter away the passion of the medical staffs. Some foreign nations have made some reimbursement for the structured education program to support it sustainable (33–35). Thus, to make the SSEP sustainable and play its role in diabetes management, the financial support for the SSEP from medical insurance or other third-party reimbursements in mainland China is required to pay the related costs (e.g., medical personnel and teaching materials).

This study had some strengths. First, this study revealed the long-term cost-effectiveness of the SSEP by exploiting the results of an actual RCT conducted in four hospitals of mainland China, so the results can effectively represent Chinese patients with T2DM. Second, the input parameters (e.g., baseline cohort characteristics, clinical effects, cost of the intervention, and other treatments) and management-related cost originated from the practical RCT in mainland China; the cost of complications was taken from Chinese published sources. The mentioned data would reveal the baseline characteristics and clinical medical practice of patients with T2DM noninsulin therapy in mainland China. Third, the univariate sensitivity analysis and the PSA were incorporated to decrease the uncertainty of simulated results.

### Limitations

Some limitations were revealed by this study. The probabilities for transitions between different states in the IQVIA CORE Diabetes Model and utilities value input were consistent with studies abroad. For this reason, it may slightly impact the study results. Secondary analysis can be conducted in the future for the presence of a simulation model and utility value based on the Chinese population. Moreover, the study was conducted based on healthcare service system in China, the direct medical cost was only considered, instead of the indirect medical cost (e.g., consumed by labor time and productivity loss). Thus, the economic assessment on the perspective of social service may be the next study interest.

### Conclusion

The SSEP is recognized as a cost-saving option for patients with T2DM with noninsulin therapy in mainland China. It is projected to downregulate the incidence of diabetes-related complications, increase QALYs, and lower direct medical cost as compared with routine education. Moreover, it is a cost-effective option for medical personnel, especially in primary hospitals, rural areas, and the regions/areas under the consistent conditions with mainland China to manage patients with T2DM.

## Data Availability Statement

The original contributions presented in the study are included in the article/Supplementary Material, further inquiries can be directed to the corresponding authors.

## Ethics Statement

The studies involving human participants were reviewed and approved by the Review Board of Peking University (IRB00001052-17031). Written informed consent was obtained from the participants of the study. The patients/participants provided their written informed consent to participate in this study.

## Author Contributions

XJ contributed to the design of the study, data collection, and analysis. LT and ML conceptualized and supervised the study. XJ, HJ, LT, and ML prepared and revised the manuscript. All the authors prepared the manuscript and approved the final version for submission.

## Funding

This study was supported by the Hainan Provincial Natural Science Foundation of China (820RC631), the Young Talents' Science and Technology Innovation Project of Hainan Association for Science and Technology (QCXM202019), the Project of Science Research Project in Hainan University of Higher Education (Hnky2020-36), the Hainan Health Commission Health Industry Research Project (20A200286), and the Key Laboratory of Tropical Cardiovascular Diseases Research of Hainan Province (KLTCDR-202002).

## Conflict of Interest

The authors declare that the research was conducted in the absence of any commercial or financial relationships that could be construed as a potential conflict of interest.

## Publisher's Note

All claims expressed in this article are solely those of the authors and do not necessarily represent those of their affiliated organizations, or those of the publisher, the editors and the reviewers. Any product that may be evaluated in this article, or claim that may be made by its manufacturer, is not guaranteed or endorsed by the publisher.
