# Supplementary material for: The Cost-Effectiveness Analysis of Self-Efficacy-Focused Structured Education Program for Patients With Type 2 Diabetes Mellitus in Mainland China Setting
Source: Front Public Health. 2021 Dec 9;9:767123. doi: 10.3389/fpubh.2021.767123 (PMC8695800; doi:10.3389/fpubh.2021.767123)
Supplement: Supplementary file 1 [file Table_1.DOC]

**Appendices S1-S5 (Supplementary data)**

| S1 Baseline cohort characteristics. | |
| --- | --- |
|  | Mean (SD) / n (%) |
| Demographic |  |
| Age, year | 56.91 (10.05) |
| Duration of T2DM, year | 6.03 (5.17) |
| Male, % | 44.9 |
| Risk factors |  |
| HbA1c, % | 8.71 (1.35) |
| Systolic pressure, mmHg | 128.31 (15.06) |
| Diastolic pressure, mmHg | 77.23 (8.71) |
| TC, mmol/L | 4.97 (1.19) |
| HDL-C, mmol/L | 1.38 (0.42) |
| LDL-C, mmol/L | 2.73 (0.83) |
| TG, mmol/L | 2.10 (1.37) |
| BMI, kg/m^2^ | 24.77 (3.38) |
| Heart rates | 77.42 (7.18) |
| Waist hip ratio | 0.943 (0.057) |
| Smoker, % | 20.8 |
| the amount of smoking, per/ day | 3.81 |
| Drinking, % | 16.6 |
| the amount of drinking, Oz/week | 1.18 |
| Cardiovascular events at baseline |  |
| Myocardial infarction | 2（0.8%） |
| Angina | 3（1.1%） |
| Stroke | 3（1.1%） |
| Heart failure | 1（0.4%） |
| Renal Diseases at baseline |  |
| Microalbuminuria | 5（1.9%） |
| Gross proteinuria | 1（0.4%） |
| Eye disease at baseline |  |
| Background diabetic retinopathy | 6（2.3%） |
| Proliferative diabetic retinopathy | 1（0.4%） |
| Cataracts | 5（1.9%） |
| foot ulcer at baseline |  |
| Uninfected ulcer | 2（0.8%） |
| Infected ulcer | 1（0.4%） |
| Neuropathy at baseline | 6（2.3%） |

†Abbreviations. SD, standard deviation; TC, total cholesterol; TG, triglycerides; LDL-C, low-density lipoprotein cholesterol; HDL-C, high-density lipoprotein cholesterol, BMI, body mass index

| S2 Cost of diabetes related complications. | | | |
| --- | --- | --- | --- |
| Complication | Year of event  (RMB) | 2+ Year  (RMB) | Source |
| Cardiovascular disease |  |  |  |
| Myocardial infarction | 73414 | 23207 | [1] |
| Angina | 35486 | 10039 | [1] |
| Congestive heart failure | 35171 | 18658 | [1] |
| Stroke | 29070 | 14381 | [1] |
| Death within 30 days due to stroke | 15887 | - |  |
| Peripheral vascular disease | 21375 | 3358 | [1] |
| Renal disease |  |  |  |
| Hemodialysis | 144238 | 116005 | [1] |
| Peritoneal dialysis | 59882 | 48842 | [2, 3] |
| Renal transplant | 259208 | 68572 | [2, 3] |
| Acute event |  |  |  |
| Non-severe hypoglycemia | 801 | - | [1] |
| Severe hypoglycemia | 12557 | - | [1] |
| Ketoacidosis | 12128 | - | [1] |
| Lactic acidosis | 8300 | - | [1] |
| Edema onset | 561 | - | [1] |
| Eye disease |  |  |  |
| Laser treatment | 6287 | - | [1] |
| Cataract operation | 10339 | 362 | [1] |
| Blindness | 2119 | 700 |  |
| Other complications |  |  | [2, 3] |
| Neuropathy | 17091 | 6557 | [2, 3] |
| Amputation | 15905 | 14283 | [2, 3] |
| Gangrene treatment | 14959 | - | [1] |
| Healed ulcer | 5402 | - | [4] |
| Infected ulcer | 23080 | - | [2, 3] |
| Uninfected ulcer | 19207 | - | [2, 3] |
| Other related management cost |  |  |  |
| Aspirin | 8.88 | - | [5] |
| Statins | 272.43 | - | [5] |
| ACE inhibitors | 17.78 | - | [5] |
| Microalbuminuria screening | 1.25 | - | [5] |
| Gross proteinuria screening | 0.75 | - | [5] |
| Eye screening | 0.41 | - | [5] |

†RMB, Ren Min Bi. Note: According to the health care consumer price index in the annual data released by the National Bureau of Statistics of China, the cost is adjusted to the year of 2017. The consumer price index of 2017, 2016, 2015, 2014, 2013, 2012 and 2011 were 106.0%, 103.8%, 102.7%, 101.7%, 101.5%, 101.7% and 102.9%, respectively.

| S3 Health state utilities inputed in the base case analysis. | | |
| --- | --- | --- |
| Health state/event | Utility | Reference |
| No complication | 0.785 | [6] |
| Myocardial event | 0.730 | [6] |
| Post-myocardial event | 0.730 | [6] |
| Angina | 0.695 | [6] |
| Congestive heart failure | 0.677 | [6] |
| Post-stroke | 0.621 | [6] |
| Peripheral vascular disease | 0.724 | [6] |
| Microalbuminuria | 0.785 | [6] |
| Gross proteinuria | 0.737 | [6] |
| Hemodialysis | 0.621 | [6] |
| Peritoneal dialysis | 0.581 | [6] |
| Renal transplant | 0.762 | [6] |
| Background diabetic retinopathy | 0.745 | [6] |
| Proliferative diabetic retinopathy | 0.715 | [6] |
| Macular oedema | 0.745 | [6] |
| Severe vision loss/blindness | 0.711 | [6] |
| Cataract | 0.769 | [6] |
| Neuropathy | 0.701 | [6] |
| Healed ulcer | 0.785 | [6] |
| Active ulcer | 0.615 | [6] |
| Amputation event | 0.505 | [6] |
| Post-amputation | 0.505 | [6] |
| Edema attack | 0.765 | [6] |
| Post-edema | 0.785 | [6] |
| Non-severe hypoglycaemic event (daytime) | 0.771 | [6] |
| Non-severe hypoglycaemic event (nocturnal) | 0.777 | [7] |
| Severe hypoglycaemic event (non treatment, daytime) | 0.767 | [8] |
| Severe hypoglycaemic event (non treatment, nocturnal) | 0.767 | [8] |
| Severe hypoglycaemic event (treatment, daytime) | 0.738 | [7] |
| Severe hypoglycaemic event (treatment, nocturnal) | 0.719 | [9] |

| S4 The cumulative incidence of diabetes-related complications between two groups (50 years). | | | |
| --- | --- | --- | --- |
| Complications | Intervention group | Control group | The difference in cumulative complications |
| Cardiovascular disease (%) |  |  | -7.75 |
| Congestive heart failure | 10.49±0.93 | 11.03±0.96 | -0.54 |
| Peripheral vascular disease onset | 16.50±1.18 | 17.55±1.13 | -1.05 |
| Angina | 9.42±0.89 | 10.72±0.99 | -1.30 |
| Stroke (onset) | 7.26±0.83 | 7.60±0.89 | -0.34 |
| Stroke (death) | 15.59±1.06 | 16.99±1.21 | -1.40 |
| Myocardial infarction | 16.99±1.14 | 19.13±1.30 | -2.14 |
| Diabetes mortality (%) | 20.71±1.24 | 21.69±1.25 | -0.98 |
| Renal disease (%) |  |  | -2.16 |
| Microalbuminuria | 42.86±1.56 | 43.95±1.60 | -1.09 |
| Gross proteinuria | 17.01±1.20 | 17.70±1.26 | -0.69 |
| End-stage renal disease | 5.78±0.74 | 6.16±0.78 | -0.38 |
| Hypoglycemia |  |  | -20.22 |
| None severe hypoglycemia | 160.89±2.77 | 181.02±2.79 | -20.13 |
| Severe hypoglycemia (non medication) | 0.68±0.03 | 0.76±0.03 | -0.08 |
| Severe hypoglycemia (medication) | 0.07±0.010 | 0.08±0.010 | -0.01 |
| Eye disease (%) |  |  | -2.01 |
| Background diabetic retinopathy | 30.35±1.44 | 31.01±1.57 | -0.66 |
| Proliferative diabetic retinopathy | 2.12±0.48 | 2.21±0.46 | -0.09 |
| Macular oedema | 27.36±1.45 | 28.21±1.48 | -0.85 |
| Severe vision loss | 13.16±1.08 | 13.57±1.08 | -0.41 |
| Cataract | 14.07±1.09 | 14.07±1.18 | -0.00 |
| Diabetic foot complications (%) |  |  | -5.27 |
| Ulcer (first time) | 50.73±1.61 | 51.80±1.59 | -1.07 |
| Recurrent ulcer | 121.19±6.32 | 124.63±6.81 | -3.44 |
| Amputation ulcer (first time) | 25.73±1.60 | 26.44±1.61 | -0.71 |
| Amputation with recurrent ulcer | 14.86±1.85 | 14.91±1.84 | -0.05 |
| Neuropathy (%) | 75.69±1.36 | 76.69±1.37 | -1.00 |

| S5 Mean lifetime cost per patient between the two groups (50 years). | | | |
| --- | --- | --- | --- |
| Costs | Intervention group  Mean | Control group  Mean | Difference  between two groups |
| Direct medical cost | 299764 | 334229 | -34465 |
| Intervention and other treatment | 84168 | 93683 | -9515 |
| Management | 1065 | 1064 | 1 |
| Complications | 214532 | 239482 | -24950 |
| Cardiovascular disease | 43765 | 47719 | -3954 |
| Renal disease | 9412 | 10550 | -1138 |
| Eye disease | 3410 | 3511 | -101 |
| None severe hypoglycemia | 74273 | 90185 | -15912 |
| Severe hypoglycemia | 5407 | 6571 | -1164 |
| Others | 78265 | 80960 | -2695 |

**References**

[1] Duan X, Li C, Li Y, Li Q. Epidemiological Characteristics, Medical Cost And Healthcare Resource Utilization Of Diabetes-related Complications Among Chinese Patients With Type 2 Diabetes Mellitus. ISPOR AP. Tokyo, Japan, 2018

[2] Wu J, He X, Liu Y. Cost-Effectiveness Analysis of Insulin Aspart 30 Versus Insulin Glargine in Patients with Type 2 Diabetes in China. Chin Pharm J. 2016: 242-247

[3] Chen X, Wei Y, Li W, Yang W, Guan H. Cost-effectiveness analysis of switch from premix human insulin to biphasic insulin aspart 30 in Chinese patients with type 2 diabetes mellitus. Chinese Journal of Diabetes. 2015; 7: 741-747

[4] Yang L, Christensen T, Sun F, Chang J. Cost-effectiveness of switching patients with type 2 diabetes from insulin glargine to insulin detemir in Chinese setting: a health economic model based on the PREDICTIVE study. VALUE HEALTH. 2012; 15: S56-S59

[5] Jiang XJ. The Clinical Effectiveness, Behavior Change Mechanism and Economic Evaluation of Structured Education in Patients with Type 2 Diabetes. Beijing: Peking University, 2019

[6] Beaudet A, Clegg J, Thuresson P, Lloyd A, McEwan P. Review of Utility Values for Economic Modeling in Type 2 Diabetes. VALUE HEALTH. 2014; 17: 462-470

[7] Goldney RD, Phillips PJ, Fisher LJ, Wilson DH. Diabetes, depression, and quality of life: a population study. DIABETES CARE. 2004; 27: 1066-1070

[8] Evans M, Khunti K, Mamdani M*, et al.*. Health-related quality of life associated with daytime and nocturnal hypoglycaemic events: a time trade-off survey in five countries. HEALTH QUAL LIFE OUT. 2013; 11: 90

[9] Marrett E, Radican L, Davies MJ, Zhang Q. Assessment of severity and frequency of self-reported hypoglycemia on quality of life in patients with type 2 diabetes treated with oral antihyperglycemic agents: A survey study. BMC research notes. 2011; 4: 251
